# Spinal infection caused by *Coxiella burnetii* and surgical treatments: a case report

**DOI:** 10.3389/fmed.2026.1785109

**Published:** 2026-04-28

**Authors:** Xu Zhai, Hong Pan, Jie Zheng

**Affiliations:** Orthopedics Department Ward 1, The 903rd Hospital of the Joint Logistic Support Force, Hangzhou, Zhejiang, China

**Keywords:** *Coxiella burnetii*, doxycycline, metagenomic next-generation sequencing, Q fever, spinal kyphosis, vertebral infection

## Abstract

Q fever is a rare global zoonosis caused by *Coxiella burnetii*, with bone and joint involvement being an uncommon manifestation that poses significant diagnostic challenges. This article reports a case of persistent focal spinal infection caused by *C. burnetii* in an elderly female without a clear epidemiological exposure history, who was initially misdiagnosed with vertebral compression fractures. The diagnosis was confirmed by third-generation nanopore-based metagenomic next-generation sequencing (mNGS), which detected 12,170 reads of *C. burnetii* with a relative abundance of 98.27%. The patient was initially treated with oral doxycycline (0.1 g q12h) and rifampin capsules (0.45 g daily) for 4 weeks, resulting in decreased inflammatory markers and reduced paravertebral abscess size. After clinical stabilization, surgical intervention (posterior approach T12–L1 vertebral lesion resection, intervertebral bone graft fusion, and pedicle screw rod fixation) was performed under general anaesthesia. Postoperative follow-up for 3 months showed a significant improvement in the patient’s low back pain [visual analogue scale (VAS) score from 6 preoperatively to 1 at 3 months] and functional status [Oswestry Disability Index (ODI) from 65% preoperatively to 10% at 3 months], with normalized inflammatory markers and a reduced *C. burnetii* IgG antibody titre (from 1:256 to 1:128). Serological follow-up revealed persistent negative IgM antibodies throughout the treatment course. This case highlights the diagnostic value of third-generation mNGS for rare spinal infections caused by *C. burnetii* and the efficacy of a multimodal treatment approach combining targeted antimicrobial therapy and surgical intervention. The rationale for antibiotic selection and surgical management is discussed, along with the limitations of the present case and clinical insights for managing similar cases.

## 1 Introduction (background)

Q fever is a rare zoonosis caused by *Coxiella burnetii*, transmitted via ruminant aerosols (1, 2). Extrapulmonary manifestations like vertebral infections are often misdiagnosed due to insidious onset and non-specific symptoms (3, 4). Osteoarticular involvement is rare, with vertebral lesions seen in over half of *C. burnetii*-associated osteoarticular infections (5). Diagnosis is hindered by non-culturable pathogens, serological limitations, and overlapping imaging with tuberculous/pyogenic spondylitis (6–10). Traditional PCR may miss low-abundance pathogens, delaying diagnosis (11).

Third-generation mNGS enables unbiased, rapid pathogen identification, overcoming culture/PCR limitations—critical for timely management of persistent *C. burnetii* infections (10–13).

*Coxiella burnetii* spondylodiscitis is extremely rare (14, 15). A recent Frontiers case (15) used mNGS to diagnose lumbar infection, with full recovery at 6 months (15). Our case is the first to use nanopore-based mNGS for this condition, reporting a multimodal approach (preoperative doxycycline-rifampin + posterior surgery) with favorable 3-month outcomes in an elderly female without classic risk factors, expanding the host spectrum.

We report an elderly female initially misdiagnosed with vertebral compression fractures, diagnosed via mNGS and managed with doxycycline-rifampin plus surgery. This case highlights mNGS’s diagnostic value, serological follow-up’s role in treatment guidance, and multimodal management efficacy, providing insights for cases where standard hydroxychloroquine therapy is inapplicable.

## 2 Case presentation

The patient, a female in her early 70s, presented to our hospital with lower back pain and limited mobility lasting 6 months following a lumbar sprain. She had no radiating pain, numbness, or paresthesia in the lower extremities, and bilateral lower extremity muscle strength was normal. Six months prior, a thoracolumbar MRI at the Affiliated Hospital of Zhejiang University suggested T12–L2 vertebral compression fracture and an old T11 vertebral compression fracture; conservative treatment with oral celecoxib capsules yielded no significant improvement, severely impairing her daily activities.

The patient had no history of chronic diseases (e.g., valvular heart disease, vascular grafts, autoimmune diseases), smoking, or alcohol consumption, and no documented contact with cattle, sheep, or other livestock/ticks. She resided in a rural area of Zhejiang Province with surrounding livestock (cattle and sheep), no travel to Q fever epidemic areas, exposure to contaminated water, or consumption of raw dairy products. Since symptom onset, she had no fever, night sweats, weight loss, saddle anaesthesia, or abnormal bowel/bladder function.

A specialized physical examination on admission (mid-July 2025) revealed thoracolumbar kyphosis and mild scoliosis with loss of normal lumbar physiological curvature, limited active waist flexion (<30°) and extension (<10°), and no erythema, swelling, warmth, or skin breakdown in the thoracolumbar region. Severe tenderness and percussion pain were noted over the T12–L1 spinous processes and paravertebral soft tissues, with no radiating pain to the lower extremities. Bilateral lower extremity skin sensation was intact (pinprick and light touch), muscle strength was Grade V (MRC scale), the straight leg raise (SLR) test was negative (<70°), and knee/ankle reflexes were normal. Bilateral hip “four” sign, Babinski sign, and Hoffmann sign were negative, with no clonus or pathological reflexes.

**Figure 1:**
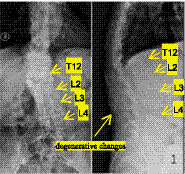
Lumbar spinal DR: Thoracolumbar scoliosis and kyphosis, degenerative changes, and osteoporosis; L2–L4 vertebral instability; localized bony lucency and sclerosis in the T12 vertebral body.

**Figure 2:**
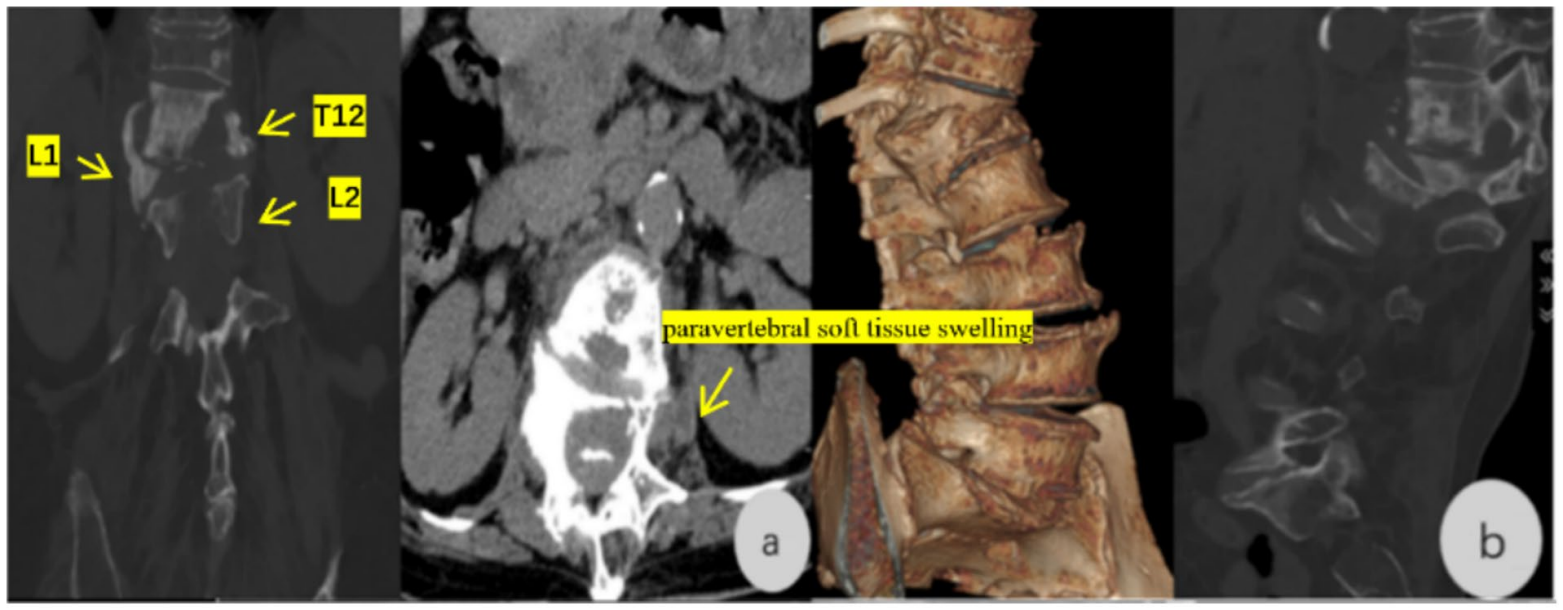
**(a,b)** Lumbar spine CT: Abnormal bone marrow signals (mixed lucency and sclerosis) in the T12–L2 vertebral bodies; paravertebral soft tissue swelling involving the ventral spinal canal.

**Figure 3:**
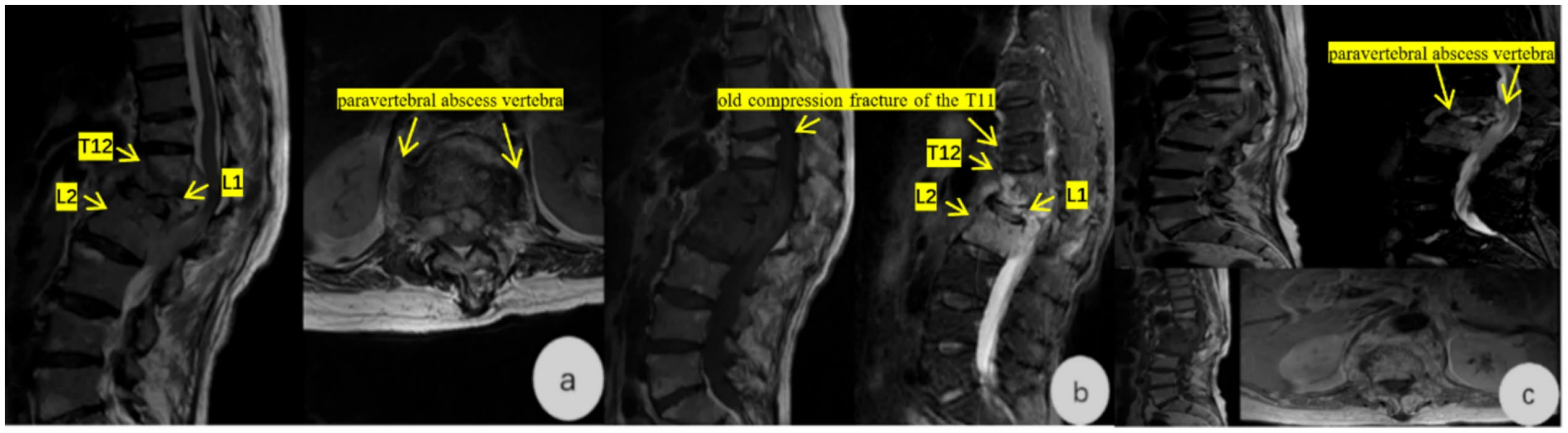
**(a,b)** Thoracolumbar spine MRI: Infectious lesions in the T12–L2 vertebral bodies with paravertebral abscess formation (dorsal and ventral); L1 vertebral body pathological fracture with mild spinal canal stenosis at the T12–L2 level; old compression fracture of the T11 vertebra. **(c)** Contrast-enhanced thoracolumbar spine MRI: Persistent infectious lesions in the T12–L2 vertebral bodies with reduced paravertebral abscess size (≈50% reduction compared with mid-June).

Imaging examinations in mid-June 2025 showed thoracolumbar DR (Figure 1): thoracolumbar scoliosis and kyphosis, degenerative changes, osteoporosis, labeled: L2–L4 vertebral instability, T11-L1 vertebral wedging, and labeled: localized bony lucency and sclerosis in T12 (Figure 1). Lumbar spine CT (Figures 2a,b): abnormal bone marrow signals in labeled: T12–L2, labeled: paravertebral soft tissue swelling ventral to the spinal canal, no sequestrum or cold abscess. Thoracolumbar MRI (Figures 3a,b): infectious lesions in labeled: T12–L2, labeled: paravertebral abscess, labeled: L1 pathological fracture, labeled: old T11 compression fracture, mild canal stenosis. Laboratory examinations in mid-June 2025 showed ESR 66 mm/h, CRP 9.94 mg/L. Serology: *C. burnetii* Phase I IgG 1:256, Phase II IgG 1:512, IgM negative. T-SPOT. TB was negative. Pathogen detection via L1 vertebral puncture used nanopore-based mNGS (MinION). Total reads: 12,389; *C. burnetii* reads: 12,170; relative abundance: 98.27%. For orthogonal validation, we performed a second targeted PCR using strain-specific primers designed within the high-read IS1111 region corresponding to the maximum read depth from mNGS. Despite this optimization, PCR remained negative, most likely due to extremely low pathogen load in the formalin-fixed, paraffin-embedded (FFPE) tissue sample. This is consistent with previous reports of low diagnostic sensitivity of PCR for chronic Q fever focal lesions. A summary table (Table 1) presents the microbial composition detected by mNGS: 98.27% of reads aligned to *C. burnetii*, with all other pathogens accounting for <1% each, confirming a single infectious etiology. Preoperative re-evaluation in mid-July 2025 showed contrast-enhanced MRI with 50% reduction in paravertebral abscess. ESR 36 mm/h, CRP 1.11 mg/L, VAS 6, ODI 65%.

**Table 1:** Results of nanopore metagenomic next-generation sequencing (mNGS).

Category	Species (common name)	Sequence reads	Sequence reads	Relative abundance (%)
Bacteria				
Gram-negative (G−)	*Citrobacter freundii*	*Citrobacter freundii*	4	0.03
Gram-positive (G+)	*Streptococcus pneumoniae*	*Streptococcus pneumoniae*	3	0.02
Viruses				
RNA virus	Human rhinovirus A	Human rhinovirus a	6	0.05
Atypical pathogens				
	*Coxiella burnetii*	*Coxiella burnetii*	12,170	98.27
Fungi	No detected pathogens			
Parasites	No detected pathogens			
Drug resistance genes	No detected resistance genes			

The patient’s treatment timeline is summarized in Table 2. After 4 weeks of anti-infective therapy with oral doxycycline (0.1 g q12h) and rifampin (0.45 g daily), she underwent surgical intervention under general anaesthesia due to thoracolumbar kyphosis, spinal canal stenosis, L1 vertebral pathological fracture, and persistent focal infection. Key surgical steps included a 16-cm midline incision from T10 to L4, dissection of bilateral sacrospinalis muscle to expose T10–L4 laminae and pedicles, bilateral insertion of 12 pedicle screws at T10, T11, T12, L2, L3, and L4 (confirmed via C-arm fluoroscopy), excision of T12–L1 supraspinous ligament, spinous processes, interspinous ligaments, and corresponding laminae for spinal canal decompression. Intraoperative exploration revealed severe L1 vertebral body destruction (only right lateral portion intact), significant paravertebral osteophytosis, and no obvious abscess/sequestrum—consistent with typical *C. burnetii* spondylitis features. Residual L1 vertebral body was resected in a V-shape via the right L1 pedicle using an ultrasonic osteotome, with thorough debridement of chronic inflammatory granulation tissue in T12–L1 and L1–L2 interspaces. Lesion tissue was collected for pathological examination and microbial culture, and the operative site was irrigated with 3,000 mL normal saline. The excised spinous process (with mild intraoperative infectious changes) was soaked in iodophor for 30 min, trimmed into strips/granules, and implanted into T12–L1–L2 interspaces for bone graft fusion. A titanium rod was placed for kyphosis correction and spinal stabilization via compression. The operation was smooth with 1,000 mL intraoperative blood loss; homologous red blood cell suspensions and plasma were transfused intraoperatively, and the patient was transferred back to the ward after anaesthesia recovery with no immediate complications.

**Table 2:** Timeline of the case.

Time point	Clinical event	Key findings	Interventions
Dec-24	Onset of lower back pain after lumbar sprain; initial diagnosis at Affiliated Hospital of Zhejiang University	MRI suggested T12–L2 vertebral compression fracture and old T11 compression fracture	Conservative treatment with oral celecoxib capsules (ineffective)
Mid-June 2025	Admission to No.903 Hospital	Thoracolumbar DR/LSD: scoliosis, kyphosis, osteoporosis, T11–L1 wedging; ESR = 66 mm/h, CRP = 9.94 mg/L; ODI = 85%; *C. burnetii* Phase I IgG = 1:256, Phase II IgG = 1:512, IgM negative	Lumbar spine puncture of L1 vertebral body; third-generation mNGS and targeted PCR for pathogen detection
Mid-June 2025	Pathogen confirmation	Third-generation mNGS: *C. burnetii* (12,170 reads, 98.27% relative abundance); targeted PCR negative; bacterial/fungal culture negative	Anti-infective therapy: oral doxycycline (0.1 g q12h) + rifampin (0.45 g daily)
Mid-July, 2025	Readmission for further treatment; clinical stabilization	Contrast-enhanced MRI: 50% reduction in paravertebral abscess size; ESR = 36 mm/h, CRP = 1.11 mg/L; VAS = 6, ODI = 65%; persistent negative *C. burnetii* IgM	Preoperative evaluation and surgical planning
Mid-July,2025	Surgical intervention	Severe L1 vertebral body destruction, no obvious abscess/sequestrum formation intraoperatively	Posterior T12–L1 lesion resection + intervertebral bone graft fusion + pedicle screw-rod fixation under general anaesthesia
Postoperative Day 1	Postoperative evaluation	Significant relief of lower back pain; normal lower extremity sensation and muscle strength (Grade V); intact bowel/bladder function	Continuation of oral doxycycline + rifampin; pain management
Postoperative Day 3	Early rehabilitation	No postoperative complications	Ambulation with thoracolumbar brace; functional exercise
Postoperative Week 2	Follow-up assessment	VAS = 4, ODI = 42%; CRP = 9.01 mg/L; ESR = 28 mm/h	Continuation of anti-infective therapy; brace-wearing for ambulation
Postoperative Month 1	Follow-up assessment	VAS = 2, ODI = 26%; normalized ESR/CRP; no imaging abnormalities	Continuation of doxycycline + rifampin; gradual increase in activity
Postoperative Month 2	Follow-up assessment	VAS = 2, ODI = 16%; stable spinal imaging; no clinical recurrence	Continuation of anti-infective therapy
Postoperative Month 3	Serological and clinical follow-up	VAS = 1, ODI = 10%; *C. burnetii* Phase I IgG = 1:128, Phase II IgG = 1:256, IgM negative; ESR/CRP within normal range; improved thoracolumbar kyphosis on imaging	Continuation of doxycycline + rifampin (planned for 12 months total); regular follow-up every 3 months

**Figure 4:**
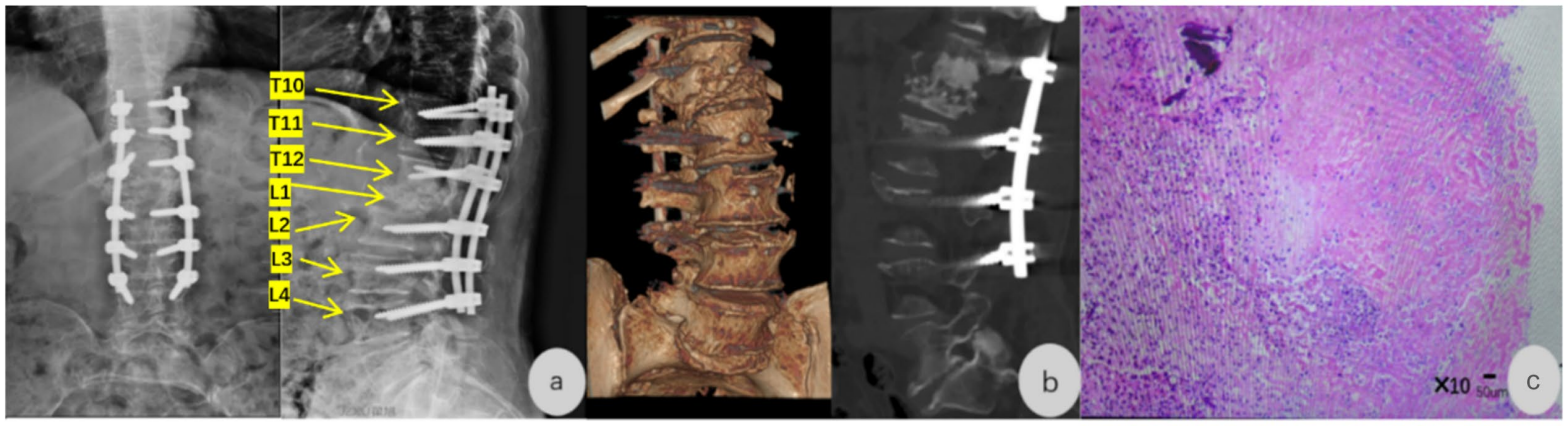
**(a,b)** Postoperative lumbar spine DR and CT: Proper positioning of pedicle screws at T10–T12 and L2–L4 levels; intact implanted bone material; no spinal canal stenosis or vertebral displacement. **(c)** Postoperative pathology: Surgical specimens of the L1 vertebral body revealed infectious lesions with fragmented fibrocartilage and a small amount of cancellous bone, accompanied by degeneration, necrosis, marked infiltration of acute and chronic inflammatory cells (neutrophils, lymphocytes, plasma cells), and fibrovascular proliferation (HE staining, ×10).

Postoperative imaging at 1 week showed significant improvement in thoracolumbar kyphosis on DR/CT (Figures 4a,b), proper pedicle screw positioning at T10-T12 and L2-L4, intact implanted bone material, and no spinal canal stenosis or vertebral displacement. Imaging features (vertebral body destruction with osteophytosis, preserved intervertebral space, small paravertebral abscess, no sequestrum) were consistent with typical *C. burnetii* spondylodiscitis reported in the literature, differing from tuberculous spondylitis (severe osteolysis, sequestrum, cold abscess, intervertebral space narrowing) and pyogenic spondylitis (rapid vertebral edge destruction, abundant pus, rapid intervertebral space narrowing)—a key reason for initial misdiagnosis. Pathological examination of L1 vertebral specimens revealed infectious lesions with fragmented fibrocartilage, small amounts of cancellous bone, degeneration, necrosis, marked infiltration of acute/chronic inflammatory cells (neutrophils, lymphocytes, plasma cells), and fibrovascular proliferation (HE staining, ×10) (Figure 4c). Special staining (acid-fast, gram, PAS) showed no acid-fast bacilli, gram-positive/negative bacteria, or fungal hyphae, and microbial culture of surgical specimens was negative for bacteria/fungi after 7 days.

Postoperative rehabilitation and follow-up showed no fever, chills, or complications (wound infection, neurological deficit, implant loosening). Anti-infective therapy with oral doxycycline (0.1 g q12h) and rifampin (0.45 g daily) was continued, with a planned total treatment duration of 12 months. The patient ambulated with a thoracolumbar brace on postoperative day 3 and received functional exercise guidance. Serial follow-up revealed progressive improvement, with detailed functional scores, inflammatory markers, and serological results summarized in Table 2. Due to the high recurrence risk of persistent focal Q fever, the patient was scheduled for long-term follow-up (every 3 months for the first 2 years, then every 6 months for the next 3 years) including clinical symptoms/functional scores (VAS/ODI), inflammatory markers (ESR/CRP), *C. burnetii* serological testing (Phase I/II IgG/IgM titres), thoracolumbar imaging (DR/CT/MRI), and echocardiography (to monitor late-onset endocarditis). At.

manuscript submission, the patient was in the 3rd month of postoperative follow-up with no recurrence signs.

### 2.1 Patient perspective

“I developed severe lower back pain after a minor lumbar sprain in December 2024, and the pain persisted for 6 months despite taking painkillers. I could barely move around and my daily life was completely disrupted. I was told I had a vertebral compression fracture, but the treatment didn’t work. After being admitted to the 903 Hospital, they found an infection in my spine and gave me medication first, then surgery. Now my pain is almost gone, and I can walk and take care of myself again. I am grateful for the accurate diagnosis and effective treatment I received.”

## 3 Discussion

Diagnosis of *C. burnetii* spinal infection is challenging due to its rarity, non-specific presentation, and limitations of traditional methods (5, 7). In this case, the patient presented with isolated lower back pain without systemic symptoms, leading to initial misdiagnosis of vertebral compression fracture—an atypical manifestation that underscores the need for heightened clinical suspicion. *Coxiella burnetii* cannot be cultured routinely, and targeted PCR was negative here due to low pathogen load, highlighting the limitations of conventional diagnostic tools; residual specimens were exhausted, precluding repeat testing. Serological testing (Phase I/II IgG/IgM) is critical to distinguish acute (elevated Phase II IgG/IgM) and persistent infection (persistent Phase I IgG with negative IgM) (7, 10), and our patient’s persistent Phase I IgG (1:256) and negative IgM supported a diagnosis of mild persistent focal infection (16). Imaging findings overlapped with tuberculous, pyogenic, and brucellar spondylitis, but we excluded these alternatives via absence of sequestrum/cold abscess (tuberculous), slow symptom progression and lack of abundant pus (pyogenic), and negative brucellosis serology. Although repeat strain-specific PCR was negative, the high-read depth (12,170 reads) and 98.27% relative abundance in mNGS, combined with consistent clinical, serological, and imaging features, confirm the diagnosis. Third-generation nanopore mNGS definitively identified *C. burnetii* as the causative pathogen, with low-abundance background flora and human rhinovirus A of no clinical significance, confirming its value for rare, difficult-to-diagnose infectious diseases (12, 13).

International guidelines recommend doxycycline + hydroxychloroquine (18 + months) for persistent Q fever, as hydroxychloroquine alkalizes host cell phagolysosomes to enhance doxycycline’s intracellular activity (7, 16, 17). This combination is based on the mechanism that hydroxychloroquine alkalizes the phagolysosome of host cells, increasing the transmembrane penetration of doxycycline and enhancing its bacteriostatic activity against the intracellular *C. burnetii* (16, 18). However, we selected doxycycline + rifampin for several key reasons: rifampin exhibits pH-independent bactericidal activity against *C. burnetii* in axenic media, in contrast to doxycycline (pH-dependent bacteriostatic activity) (16, 17). Prior to the widespread use of the doxycycline-hydroxychloroquine regimen, rifampin + doxycycline was the first-line therapy for Q fever endocarditis and persistent focal infection, with favourable clinical outcomes (19–21). Rifampin also has good tissue penetration (including bone and spinal tissue), which is critical for the treatment of spinal infection (20). Additionally, our elderly rural patient could not comply with hydroxychloroquine’s required ocular and electrolyte monitoring, while rifampin’s once-daily dosing and liver function monitoring were more feasible in her clinical setting. The 4-week preoperative course yielded a rapid clinical response—reduced inflammation, shrunk abscess size, and clinical stabilization—confirming the regimen’s efficacy, and rifampin was well-tolerated with no adverse events.

We plan a 12-month total treatment duration, shorter than the guideline-recommended 18 months, based on the patient’s rapid clinical/serological response and surgical debridement of focal infection. Given the single-case nature of this report and the insufficient follow-up period, we acknowledge that this treatment duration cannot be generalized as a standard. For this specific patient, we plan to extend antibiotic therapy beyond 12 months if any of the following are observed during long-term follow-up: (1) failure of *C. burnetii* Phase I IgG titre to decrease by ≥50% from baseline (preoperative Phase I IgG = 1:256) at 6 months postoperatively; (2) persistent elevation of inflammatory markers (ESR > 20 mm/h or CRP > 8 mg/L) without alternative explanations (e.g., other infections, inflammatory diseases); (3) radiological evidence of residual or recurrent infection (e.g., persistent vertebral lesions, recurrent paravertebral abscess, progressive vertebral destruction) on thoracolumbar DR/CT/MRI; or (4) clinical recurrence of symptoms (e.g., recurrent lower back pain, fever, neurological deficits). We emphasize that this monitoring plan reflects the individualized management of this specific patient and should not be interpreted as a generalizable protocol or clinical standard. Long-term follow-up will closely monitor for recurrence to guide any necessary adjustments to therapy.

A recent Frontiers case by Tan et al. (22) reported lumbar *C. burnetii* spondylodiscitis managed with L5-S1 decompression/fusion and long-term doxycycline/ciprofloxacin, with full recovery at 6 months. Our case differs in several key aspects: we used nanopore-based mNGS (faster turnaround, portability) for diagnosis versus second-generation Illumina mNGS (12), selected doxycycline + rifampin (bactericidal, superior bone penetration) over doxycycline + ciprofloxacin (16, 19), performed T10-L4 long-segment fixation + T12-L1 lesion resection/kyphosis correction (addressing thoracolumbar destruction and instability) versus single-segment decompression, and involved an elderly female without clear epidemiological exposure versus a middle-aged male with livestock contact—expanding the known host spectrum of the disease.

Our multimodal treatment approach proved effective: preoperative antibiotics reduced inflammation and minimized surgical risk (16), while posterior debridement, decompression, and stabilization resolved pain and restored function—consistent with *C. burnetii* spondylitis’s typical intraoperative features (no abscess/sequestrum) (5, 14). Postoperatively, long-term antibiotics and serial serological follow-up were critical to prevent recurrence, with declining Phase I/II IgG titres indicating a favorable therapeutic response (10, 16). The strengths of this case include the first use of nanopore-based mNGS for *C. burnetii* spinal infection (12, 13), a novel multimodal treatment strategy, and comprehensive evaluation excluding alternative diagnoses (7, 10). Limitations include a 3-month follow-up period (short for persistent Q fever, which requires prolonged monitoring) (7, 16), single-case design, Phase I IgG titre <1:1024 (below Dutch chronic Q fever diagnostic criteria) (9, 10), and no use of hydroxychloroquine (17); long-term follow-up and larger multicenter studies are needed to validate our approach.

For clinicians managing suspected *C. burnetii* spinal infection, several key insights emerge. First, maintain high suspicion for *C. burnetii* in patients with persistent back pain and non-specific imaging, especially in rural areas with livestock exposure (3, 15). Second, use third-generation mNGS early when traditional diagnostics are inconclusive, as it overcomes limitations of culture and targeted PCR for rare pathogens (12, 13). Third, perform complete *C. burnetii* serology (Phase I/II IgG/IgM) and serial follow-up to distinguish acute/persistent infection and guide treatment duration (7, 10). Fourth, individualize antibiotic therapy: doxycycline + rifampin is a viable alternative to doxycycline + hydroxychloroquine when ocular/electrolyte monitoring is impractical, with clear thresholds for treatment extension (16, 19). Fifth, consider surgical intervention for spinal instability or vertebral destruction after preoperative antibiotic stabilization to resolve mechanical symptoms and eliminate focal infection (5, 22). Finally, conduct long-term follow-up (≥2 years) to monitor for recurrence, using serological, inflammatory, and radiological markers to assess treatment response (7, 16).

## 4 Conclusion

This case report describes the first use of third-generation nanopore-based mNGS to diagnose *C. burnetii* spinal infection in an elderly female, successfully managed with preoperative doxycycline-rifampin therapy followed by posterior spinal surgery. It highlights mNGS’s diagnostic value for rare infections, serological follow-up’s role in treatment guidance, and the efficacy of individualized multimodal management—including a patient-specific monitoring plan for antibiotic duration adjustment. Given the single-case design and limited follow-up period, the 12-month antibiotic duration reported here is specific to this patient’s clinical response and cannot be generalized as a standard. While limited by short follow-up and single-case design, this report provides practical clinical insights for cases where standard hydroxychloroquine therapy is inapplicable, enriching the evidence base for *C. burnetii*-associated spinal infections and offering actionable guidance for clinicians.

## Data Availability

The datasets presented in this article are not readily available because of ethical and privacy restrictions. Requests to access the datasets should be directed to the corresponding author.

## References

[1] Gagnier JJ Kienle G Altman DG Moher D Sox H Riley D . The CARE guidelines: consensus-based clinical case reporting guideline development. Headache. (2013) 53:1541–7. doi: 10.1111/head.12246, 24228906 PMC3844611

[2] Bae M Jin CE Park JH . Diagnostic usefulness of molecular detection of *Coxiella burnetii* from blood of patients with suspected acute Q fever. Medicine (Baltimore). (2019) 98:e15724. doi: 10.1097/MD.0000000000015724, 31169672 PMC6571429

[3] Million M Raoult D. Recent advances in the study of Q fever epidemiology, diagnosis and management. J Infect. (2015) 71:S2–9. doi: 10.1016/j.jinf.2015.04.024 25917809

[4] Kampschreur LM Oosterheert JJ de Vries Feyens CA Delsing CE Hermans MHA van Sluisveld ILL . Chronic Q fever-related dual pathogen endocarditis: case series of three patients. J Clin Microbiol. (2011) 49:1692–4. doi: 10.1128/JCM.02596-10, 21289146 PMC3122851

[5] Alder KD Fiegen AP Rode MM . Chronic Q fever presenting as bilateral extensor tenosynovitis: a case report and review of the literature. J Bone Jt Infect. (2023) 8:39–44. doi: 10.5194/jbji-8-39-2023, 36756305 PMC9901517

[6] Kampschreur LM Wegdam-Blans MC Wever PC Wegdam-Blans MCA Renders NHM Delsing CE . Chronic Q fever diagnosis: consensus guideline versus expert opinion. Emerg Infect Dis. (2015) 21:1183–8. doi: 10.3201/eid2107.130955, 26277798 PMC4480373

[7] Eldin C Mélénotte C Mediannikov O Ghigo E Million M Edouard S . From Q fever to *Coxiella burnetii* infection: a paradigm change. Clin Microbiol Rev. (2017) 30:115–90. doi: 10.1128/CMR.00045-16, 27856520 PMC5217791

[8] Wegdam-Blans MC Vainas T van Sambeek MR . Vascular complications of Q-fever infections. Eur J Vasc Endovasc Surg. (2011) 42:384–92. doi: 10.1016/j.ejvs.2011.04.013 21622013

[9] Raoult D. Chronic Q fever: expert opinion versus literature analysis and consensus. J Infect. (2012) 65:102–8. doi: 10.1016/j.jinf.2012.04.006, 22537659

[10] Wegdam-Blans MC Kampschreur LM Delsing CE Wegdam-Blans MCA Bleeker-Rovers CP Sprong T . Chronic Q fever: review of the literature and a proposal of new diagnostic criteria. J Infect. (2012) 64:247–59. doi: 10.1016/j.jinf.2011.12.014 22226692

[11] Fournier PE Marrie TJ Raoult D. Diagnosis of Q fever. J Clin Microbiol. (1998) 36:1823–34. doi: 10.1128/JCM.36.7.1823-1834.1998 9650920 PMC104936

[12] Zhao M Tang K Liu F Zhou W Fan J Yan G . Metagenomic next-generation sequencing improves diagnosis of osteoarticular infections from abscess specimens: a multicenter retrospective study. Front Microbiol. (2020) 11:2034. doi: 10.3389/fmicb.2020.02034, 33042033 PMC7523410

[13] Huang ZD Zhang ZJ Yang B . Pathogenic detection by metagenomic next-generation sequencing in osteoarticular infections. Front Cell Infect Microbiol. (2020) 10:471. doi: 10.3389/fcimb.2020.00471, 33042860 PMC7527540

[14] Meriglier E Sunder A Elsendoorn A . Osteoarticular manifestations of Q fever: a case series and literature review. Clin Microbiol Infect. (2018) 24:912–3. doi: 10.1016/j.cmi.2018.03.005, 29549057

[15] Song M Guo Y Hao J Zheng C Zuo H Ye J . Case report: *Coxiella burnetii* vertebral osteomyelitis in a pigeon breeder: mNGS diagnosis of chronic Q fever. Front Med. (2025) 12:1636778. doi: 10.3389/fmed.2025.1636778, 40959427 PMC12434005

[16] Smith CB Evavold C Kersh GJ. The effect of pH on antibiotic efficacy against *Coxiella burnetii* in axenic media. Sci Rep. (2019) 9:18132. doi: 10.1038/s41598-019-54556-6, 31792307 PMC6889355

[17] Delahaye A Eldin C Bleibtreu A Djossou F Marrie TJ Ghanem-Zoubi N . Treatment of persistent focalized Q fever: time has come for an international randomized controlled trial. J Antimicrob Chemother. (2024) 79:1725–47. doi: 10.1093/jac/dkae145, 38888195

[18] España PP Uranga A Cillóniz C Torres A. Q Fever (*Coxiella Burnetii*). Semin Respir Crit Care Med. (2020) 41:509–21. doi: 10.1055/s-0040-1710594, 32629489

[19] Botelho-Nevers E Fournier PE Richet H Fenollar F Lepidi H Foucault C . *Coxiella burnetii* infection of aortic aneurysms or vascular grafts: report of 30 new cases and evaluation of outcome. Eur J Clin Microbiol Infect Dis. (2007) 26:635–40. doi: 10.1007/s10096-007-0357-6, 17629755

[20] Bental T Fejgin M Keysary A Rzotkiewicz S Oron C Nachum R . Chronic Q fever of pregnancy presenting as *Coxiella burnetii* placentitis: successful outcome following therapy with erythromycin and rifampin. Clin Infect Dis. (1995) 21:1318–21. doi: 10.1093/clinids/21.5.1318, 8589167

[21] Hellmeyer L Schmitz-Ziegler G Slenczka W . Q fever in pregnancy: a case report and review of the literature. Z Geburtshilfe Neonatol. (2002) 206:193–8. doi: 10.1055/s-2002-34961, 12395293

[22] Tan X Li F Zhang T . *Coxiella burnetii* infection in the lumbar vertebra: a rare case report and review of literature. Front Med (Lausanne). (2025) 12:1618670. doi: 10.3389/fmed.2025.1618670, 40823585 PMC12354406

